# Phase I study of Nivolumab, an anti-PD-1 antibody, in patients with malignant solid tumors

**DOI:** 10.1007/s10637-016-0411-2

**Published:** 2016-12-08

**Authors:** Noboru Yamamoto, Hiroshi Nokihara, Yasuhide Yamada, Takashi Shibata, Yosuke Tamura, Yoshitaka Seki, Kazunori Honda, Yuko Tanabe, Hiroshi Wakui, Tomohide Tamura

**Affiliations:** 1 Department of Thoracic Oncology, National Cancer Center Hospital, 5-1-1 Tsukiji Chuo-ku, Tokyo, 104-0045 Japan; 2 Department of Gastrointestinal Oncology, National Cancer Center Hospital, 5-1-1 Tsukiji Chuo-ku, Tokyo, 104-0045 Japan

**Keywords:** Phase I study, Nivolumab, Immune checkpoint inhibitor, Pharmacokinetics

## Abstract

*Background* This study evaluated the safety, tolerability, pharmacokinetics, immunogenicity and antitumor activity of single and multiple doses of nivolumab in Japanese patients with malignant solid tumors. *Subjects and Methods* This was an open-label, dose-escalation study in 17 patients with advanced solid tumors with a life expectancy of ≥3 months. Patients were observed for 3 weeks after a single dose of nivolumab at 1, 3, 10 or 20 mg/kg, then received the same dose of nivolumab every 2 weeks until unacceptable toxicity or disease progression occurred. This study included a maximum dose of 20 mg/kg, which is the highest dose of nivolumab evaluated to date. The maximum dose was 10 mg/kg in previous studies. *Results* The commonest adverse drug reaction was lymphopenia, which occurred in 10 (58.8%) patients, including two (11.8%) with Grade ≥3 events. No dose-limiting toxicities (DLTs) were observed up to the maximum dose of 20 mg/kg. The area under the concentration–time curve from time 0 to the last measurable concentration was linear up to 20 mg/kg. The maximum concentration showed dose-dependency up to 10 mg/kg, but not between 10 and 20 mg/kg. One durable complete response and two partial responses were observed. *Conclusions* Nivolumab at doses of 1–20 mg/kg was not associated with DLTs, and it was generally well tolerated at doses of up to 20 mg/kg in Japanese patients with advanced solid tumors.

**Electronic supplementary material:**

The online version of this article (doi:10.1007/s10637-016-0411-2) contains supplementary material.

## Introduction

Cancer accounted for 330,000 deaths in Japan—approximately 30% of all deaths—in 2010 [1]. Despite advances in cancer treatment, the morbidity and mortality rates associated with some types of cancer are expected to continue to increase [2].

Immunotherapeutic agents, such as biologics, which use the body’s own immunity to eliminate tumors or directly target tumors, have been under investigation for several decades. In recent years, these agents have been included alongside surgery, radiation and chemotherapy in the treatment of many types of cancer [3, 4]. More recently, vaccine therapies using cancer antigens or dendritic cells have been studied, but their response rates were as low as 2.6%, partly because of the expression of molecules (e.g., cytokines and co-stimulators) that suppress immunological activity on regulatory lymphocytes and cancer cells themselves [5].

Programmed death-1 (PD-1) (also known as CD279) is a CD28 family receptor that is expressed on activated lymphocytes (T cells, B cells and NKT cells) and myeloid cells. PD-1 and its ligands defend against self-reactive effector T cells by promoting the development and function of regulatory T cells and directly inhibiting potentially pathogenic self-reactive peripheral T cells [6]. PD-1 ligands are expressed on antigen-presenting cells (APCs) and parenchymal cells. PD-1 ligands on APCs induce immunological tolerance by switching off autoreactive T cells, while ligands expressed on parenchymal cells suppress effector T cells, thus maintaining peripheral tolerance by preventing tissue destruction [7]. PD-1, its ligands (e.g., PD-L1), or both are overexpressed in many autoimmune diseases, including inflammatory bowel disease, rheumatoid arthritis and type 1 diabetes [6].

PD-1 deficiency in mice leads to the development of various autoimmune disorders, such as dilated cardiomyopathy associated with autoantibody production in BALB/c mice, systemic lupus erythematosus-like glomerular nephritis and arthritis in C57BL/6 mice, myocarditis associated with generation of autoantibody in MRL mice, and accelerated type 1 diabetes in non-obese diabetic mice, suggesting that PD-1 is involved in the suppression of autoimmune reactions [7, 8].

PD-L1 expression by tumor cells may induce and maintain regulatory T cells in the tumor, allowing tumor progression by enhanced suppression of antitumor T-cell responses [9]. In addition to APCs, PD-1 ligands are expressed in various human cancer tissues [9], and PD-L1 expression in resected cancer tissues was correlated with postoperative survival time in ovarian cancer [10], malignant melanoma [11], esophageal cancer [12], renal cell carcinoma (RCC) [13], pancreatic cancer [14], and urothelial cancer [15], for example. In an experiment using a murine cancer cell line, the PD-1 ligand-knockout murine cancer cell line (P815 cells) was attacked by cytotoxic T cells specific to this cancer cell line. Transfection of this cell line with PD-L1 decreased the cells’ susceptibility to cytotoxic T cells. In an autologous tumor-bearing model using the same PD-L1–transfected cancer cell line, proliferation of the transplanted cancer cells was inhibited by anti–PD-1 antibody [16].

These findings suggest that the PD-1/PD-1 ligand pathway is involved in cancer cell immunity, and that PD-1 inhibitors are expected to be useful anticancer agents [7].

Nivolumab (Ono Pharmaceutical developmental code: ONO-4538; Bristol-Myers Squibb [BMS] developmental code: BMS-936558; ex-Medarex developmental code: MDX-1106) is a fully human monoclonal antibody to human PD-1 that was co-developed by Ono Pharmaceutical Co., Ltd. and Medarex Inc.

Nivolumab was well tolerated when administered in a single dose at up to 10 mg/kg, with a mean half-life (*t*_1/2_) of 17 to 24.8 days in an early Phase I study (unpublished data: ClinicalTrials.gov [NCT00441337]). Phase II and III studies have shown promising efficacy and tolerability of nivolumab in advanced melanoma [17], refractory squamous non-small cell lung cancer (NSCLC) [18], metastatic RCC [19], platinum-resistant ovarian cancer [20], and Hodgkin’s lymphoma [21], for example.

The objectives of the present study were to evaluate the safety, tolerability, and pharmacokinetics of single or multiple doses of nivolumab in Japanese patients with malignant solid tumors, and to investigate the pharmacological activity and efficacy of nivolumab.

## Methods

### Patient selection

The study enrolled patients with histologically or cytologically confirmed malignant solid tumors for whom standard therapy was ineffective or for whom no appropriate treatment was available. The inclusion criteria included age ≥20 years; Eastern Cooperative Oncology Group (ECOG) performance status (PS) 0 or 1; measurable lesion; life expectancy of ≥3 months; and adequate organ functions (AST ≤82.5 IU/L; ALT ≤105 IU/L; total bilirubin ≤2.4 mg/dL; creatinine ≤2.0 mg/dL).

Chemotherapy, immunotherapy (e.g., tumor vaccines and cytokines), surgery, and radiotherapy were prohibited for 4 weeks prior to study treatment. Radiopharmaceuticals (e.g., strontium) were prohibited for 8 weeks prior to study treatment. No patients with primary or metastatic lesions in the brain or meninges were included unless they were asymptomatic and did not require treatment. Women of childbearing potential and men were required to use contraception.

The major exclusion criteria included: history of severe hypersensitivity reaction to other antibody products; adverse drug reactions (ADRs) associated with prior treatments or surgical therapy with a possible influence on the study outcome; multiple primary cancers; any active autoimmune disease or documented history of any chronic or recurrent autoimmune disease or requirement for systemic steroids or immunosuppressants; requirement for or a history of transplantation; and failure of a fixed-dose narcotic analgesic regimen to control bone pain associated with bone metastases.

### Study design

**Fig. 1:**
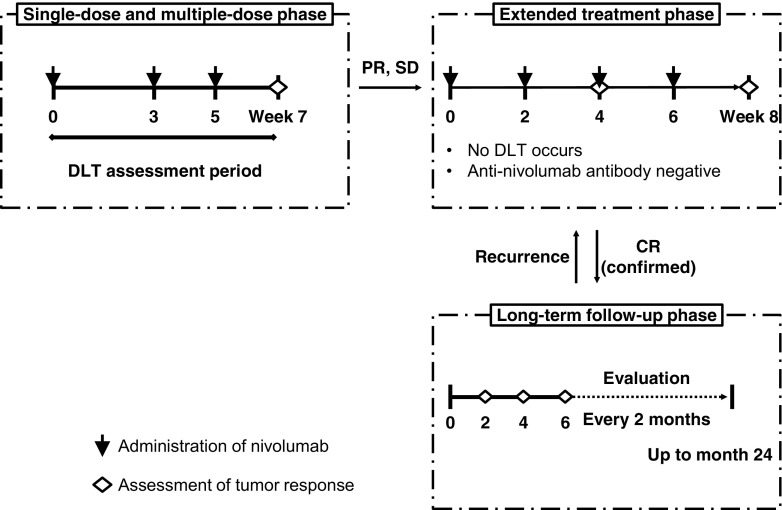
Study design. *DLT* dose-limiting toxicity, *PR* partial response, *SD* stable disease

The study was a single-center, open-label dose-escalation study (Fig. 1). The dose-escalation methodology was conducted as follows. Patients were observed for 3 weeks after a single dose of nivolumab. Eligible patients continued to receive nivolumab at the initial dose every 2 weeks until disease progression or unacceptable toxicities were observed (up to 24 months). The dosing regimen was based on a previous phase I single-dose study in which the mean *t*_1/2_ was 17 to 24.8 days for nivolumab at doses of 0.3 to 10 mg/kg (unpublished data: ClinicalTrials.gov [NCT00441337]).

Dose-limiting toxicity (DLT) was defined as any adverse event (AE) occurring up to 7 weeks after the first nivolumab treatment that fulfilled any of the following criteria and for which a causal relationship with the study drug could not be ruled out:

- Grade 4 neutropenia persisting for 7 days,
- Grade 4 thrombopenia or Grade 3 thrombopenia requiring blood transfusion,
- Grade ≥3 non-hematological toxicities, except for the following toxicities:

- Grade 4 flu-like syndrome,
- Grade ≥3 nausea, vomiting, anorexia or diarrhea that does not resolve despite full supportive care, or,
- Grade ≥3 hyponatremia, fever, or injection site reaction that in the investigator’s or subinvestigator’s opinion requires study discontinuation.

**Fig. 2:**
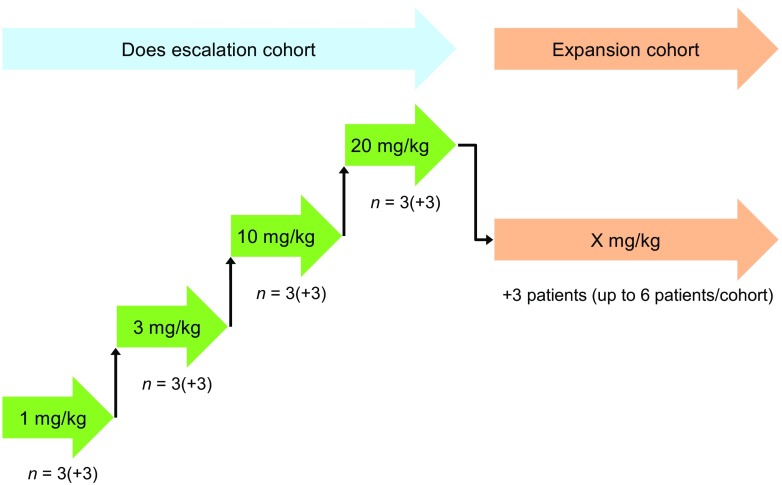
Dose escalation and dose-limiting toxicities

The methodology relating to dose-escalation and DLTs is shown in Fig. 2. If a DLT was observed in 0/3 patients or only 1/6 patients in a step, patients could be enrolled into the next dose step.

All ADRs were evaluated using NCI-CTCAE ver.3.

Serum concentrations of nivolumab were measured using a validated enzyme-linked immunosorbent assay (ELISA), which was based on a sandwich ELISA platform using a recombinant human PD-1/Fc chimeric protein as a capture and mouse anti-human IgG4-alkaline phosphatase as a detector. Serum samples were collected prior to dosing, just before the end of the nivolumab infusion and at 2 h and 8 h post-dose, on days 2, 3, 4, 8, 15 and 22, and at study discontinuation.

### Administration of nivolumab

The study drug administered throughout this trial was nivolumab (ONO-4538 Intravenous Infusion 100 mg). Patients received their assigned dose of nivolumab for the duration of the study (1, 3, 10 or 20 mg/kg), infused intravenously over 1 h, through a 0.2-μm in-line filter in a total volume of 60 mL.

### Objectives

The study was designed to evaluate the safety, tolerability, and pharmacokinetics of nivolumab in Japanese patients with malignant solid tumors, and to investigate the pharmacological activity and efficacy of nivolumab. The primary objective was to assess the safety and tolerability of nivolumab in terms of ADRs, DLTs, laboratory parameters and vital signs. We intended to use this information to estimate the maximum tolerated dose (MTD) of nivolumab for use in future studies. The efficacy of nivolumab was assessed as an exploratory endpoint in terms of the tumor’s response (as measured by diagnostic imaging) and changes in tumor markers. The tumor responses were assessed according to the RECIST guideline version 1.0. The pharmacokinetics of nivolumab were assessed using the serum nivolumab concentration. As exploratory endpoints, we also investigated the following pharmacodynamic parameters: lymphocyte subset analysis (CD3/CD4/CD8/CD19/CD56/CD45RO/HLA-DR/PD-1); cellular immune response (CEF peptide antigen; an antigen in which peptides derived from cytomegalovirus antigen, Epstein Barr (EB) virus antigen, and influenza virus antigen are mixed), humoral immune response (anti-NY-ESO-1, anti-survivin and anti-p53); serum cytokines (IL-2, IL-5, IL-6, IL-8, IL-10, IFN-g, TNF-α, TGF-β); immunoglobulin (antibody) (IgM, IgG1, IgG2, IgG3, IgG4 and IgA); and tumor biopsy (optional).

The anti-nivolumab antibody titer was measured by electrochemiluminescence. Immunohistochemical analysis for PD-L1 was performed with anti-human CD274/PD-L1 antibody (clone: 27A2) (Medical & Biological Laboratories Co., Ltd., Nagoya, Japan).

### Data analysis

Four study populations were defined for data analysis. The safety population comprised all patients with the target disease who received nivolumab at least once. The pharmacokinetic population comprised all patients compliant with good clinical practice [GCP] criteria who received nivolumab and had sufficient evaluable pharmacokinetic data. The pharmacodynamic and efficacy populations comprised all patients compliant with GCP criteria who received nivolumab and had evaluable efficacy or pharmacodynamic data.

DLTs were evaluated during the 7-week period after the first dose of nivolumab. ADRs (AEs for which a causal relationship with nivolumab could not be ruled out) were analyzed from the start to the end of the clinical study, and were tabulated by system organ class, preferred term and grade. The numbers of deaths, numbers of patients with serious ADRs, and numbers of patients with ADRs leading to treatment discontinuation were also tabulated.

Pharmacokinetic parameters (maximum concentration [C_max_], time to the maximum concentration [*t*_max_], area under the concentration–time curve from time 0 to the last measurable concentration [AUC_last_] and *t*_1/2_) were also analyzed.

For the pharmacodynamic analysis, the results of the lymphocyte subset analysis, antigen immune reactivity assays using peripheral blood mononuclear cells, serum cytokine and immunoglobulin (antibodies) levels, and tumor biopsy were listed by patient after the multiple-dose phase and from the start to the end of clinical study.

The efficacy parameters assessed at the end of the clinical study were analyzed as follows. The frequency distributions of best overall responses were plotted by dose step, and the numbers and percentages of responders were calculated. The time to progression, progression-free survival, duration of complete response (CR), duration of response, time to CR, and time to response were listed by patient. No statistical hypothesis tests were performed. The overall sample size and the number of subjects per dosage were calculated in reference to the Guidelines for Methods of Clinical Evaluation of Antimalignant Tumor Drugs (MHLW Notification No. 1101001 of the Evaluation and Licensing Division, PFSB dated November 1, 2005) [22], where at least three subjects per dose were needed to consider moving to the next dose.

## Results

### Patients

Seventeen patients were enrolled from September 2008 to September 2010, and all were included in the efficacy, safety, and pharmacokinetic analyses. There were three patients in the 1 mg/kg group, five patients in the 3 mg/kg group, six patients in the 10 mg/kg group, and three patients in the 20 mg/kg group.

The baseline characteristics of the study participants are shown in Table 1. There were 10 males and 7 females. The median (range) age of all patients was 61.0 (34–74) years. The cancer types are listed in Table 1. Patients had previously received surgery (70.6%), radiotherapy (29.4%), chemotherapy (94.1%), molecular-targeted therapy (52.9%), immunotherapy (29.4%), and endocrine therapy (23.5%).

**Table 1:** Baseline characteristics of patients

	Nivolumab			
	1 mg/kg (*n* = 3)	3 mg/kg (*n* = 5)	10 mg/kg (*n* = 6)	20 mg/kg (*n* = 3)
Male	2	0	6	2
Female	1	5	0	1
Median age (range)	68.0 (61–74)	55.0 (34–73)	50.0 (35–74)	68.0 (64–71)
Performance status (ECOG)				
0	1	1	2	0
1	2	4	4	3
Cancer type				
Lung adenocarcinoma	1	2	2	0
Rectal cancer	1	1	0	1
Thymic carcinoma	0	0	2	0
Esophageal carcinoma	0	0	0	1
Melanoma	1	2	1	0
Colon cancer	0	0	0	1
Thyroid cancer	0	0	1	0
Previous treatment				
Surgery	3	4	4	1
Radiotherapy	0	2	2	1
Chemotherapy	3	5	5	3
Molecular targeted therapy	2	2	3	2
Immunotherapy	1	3	1	0
Endocrine therapy	1	2	1	0

Data are n (%) unless otherwise indicated

ECOG Eastern Cooperative Oncology Group

### Safety

ADRs occurring in two or more patients are shown in Table 2. No DLTs were observed up to the highest dose of 20 mg/kg.

**Table 2:** Adverse drug reactions occurring in two or more patients by nivolumab dose and grade

	Nivolumab							
	1 mg/kg (*n* = 3)		3 mg/kg (*n* = 5)		10 mg/kg (*n* = 6)		20 mg/kg (*n* = 3)	
SOC and PT^a^	Grade ≥3	All grades	Grade ≥3	All grades	Grade ≥3	All grades	Grade ≥3	All grades
Overall	0	3	1	5	0	6	1	3
Cardiac disorders								
Ventricular extrasystoles	0	0	0	1	0	2	0	1
Gastrointestinal disorders								
Constipation	0	0	0	1	0	2	0	0
Diarrhea	0	0	0	1	0	1	0	0
General disorders and administration site conditions								
Fatigue	0	0	0	1	0	2	0	1
Malaise	0	0	0	0	0	2	0	0
Pyrexia	0	1	0	1	0	2	0	2
Investigations								
Alanine aminotransferase increased	0	1	0	0	0	1	0	0
Blood albumin decreased	0	0	0	1	0	2	0	2
Blood creatinine increased	0	0	0	0	0	2	0	0
Blood lactate dehydrogenase increased	0	0	0	0	0	2	0	0
Blood thyroid stimulating hormone increased	0	0	0	1	0	1	0	0
Blood urea increased	0	0	0	0	0	1	0	1
Blood uric acid increased	0	0	0	0	0	3	0	1
C-reactive protein increased	0	0	0	1	0	1	0	0
Eosinophil count increased	0	2	0	3	0	2	0	1
Hematocrit decreased	0	0	0	1	0	2	0	0
Hemoglobin decreased	0	0	0	1	0	2	0	0
Lymphocyte count decreased		1	1	2		5	1	2
Protein total decreased	0	0	0	1	0	0	0	1
White blood cell count increased	0	1	0	0	0	1	0	1
Tri-iodothyronine free decreased	0	0	0	0	0	1	0	1
Rheumatoid factor increased	0	0	0	1	0	1	0	0
Metabolism and nutrition disorders								
Decreased appetite	0	0	0	0	0	2	0	1
Skin and subcutaneous tissue disorders								
Erythema	0	0	0	1	0	1	0	0
Pruritus	0	0	0	1	0	2	0	0
Rash	0	1	0	2	0	2	0	0

*SOC* system organ class, *PT* preferred term

^a^MedDRA SOC/PT classification. Adverse drug reactions constituted adverse events for which a causal relationship with nivolumab could not be ruled out

The commonest ADR was lymphopenia, which occurred in 10 (58.8%) patients, including two (11.8%) with Grade ≥3 events. The other common ADRs included eosinophilia in eight patients (47.1%); pyrexia in six patients (35.3%); reduced blood albumin and rash in five patients each (29.4%); and ventricular extrasystoles, fatigue and increased blood uric acid in four patients each (23.5%). One ADR (dehydration in one patient in the 10 mg/kg group) was classified as serious.

There were no treatment-related deaths from the first dose of the study drug to the end of the follow-up period.

During the period from the start to the end of clinical study, no abnormal change in vital signs was observed in any subject. Clinically significant electrocardiographic abnormalities were observed in six patients, but no clinically significant abnormalities in chest X-ray were observed.

#### Safety according to anti-nivolumab antibody status

Anti-nivolumab antibody tests measured between the start and the end of the study were positive in 2/17 patients. There were no serious allergic reactions in any patients.

### Pharmacokinetics

**Fig. 3:**
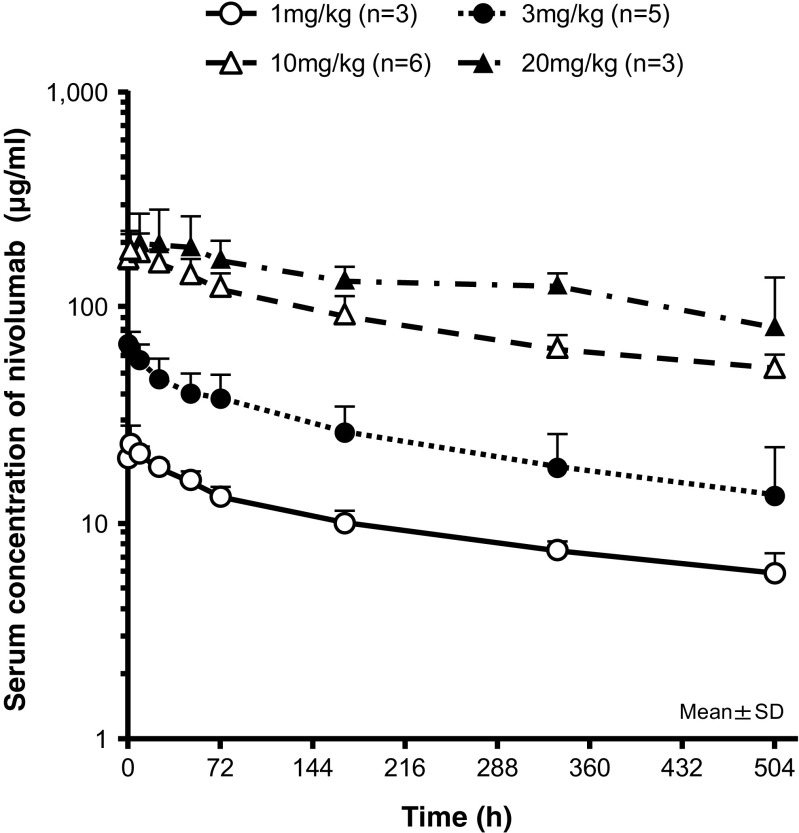
Mean serum concentration-time profiles of nivolumab after single intravenous infusion over 1 h to Japanese patients with malignant tumors at doses of 1 to 20 mg/kg

**Fig. 4:**
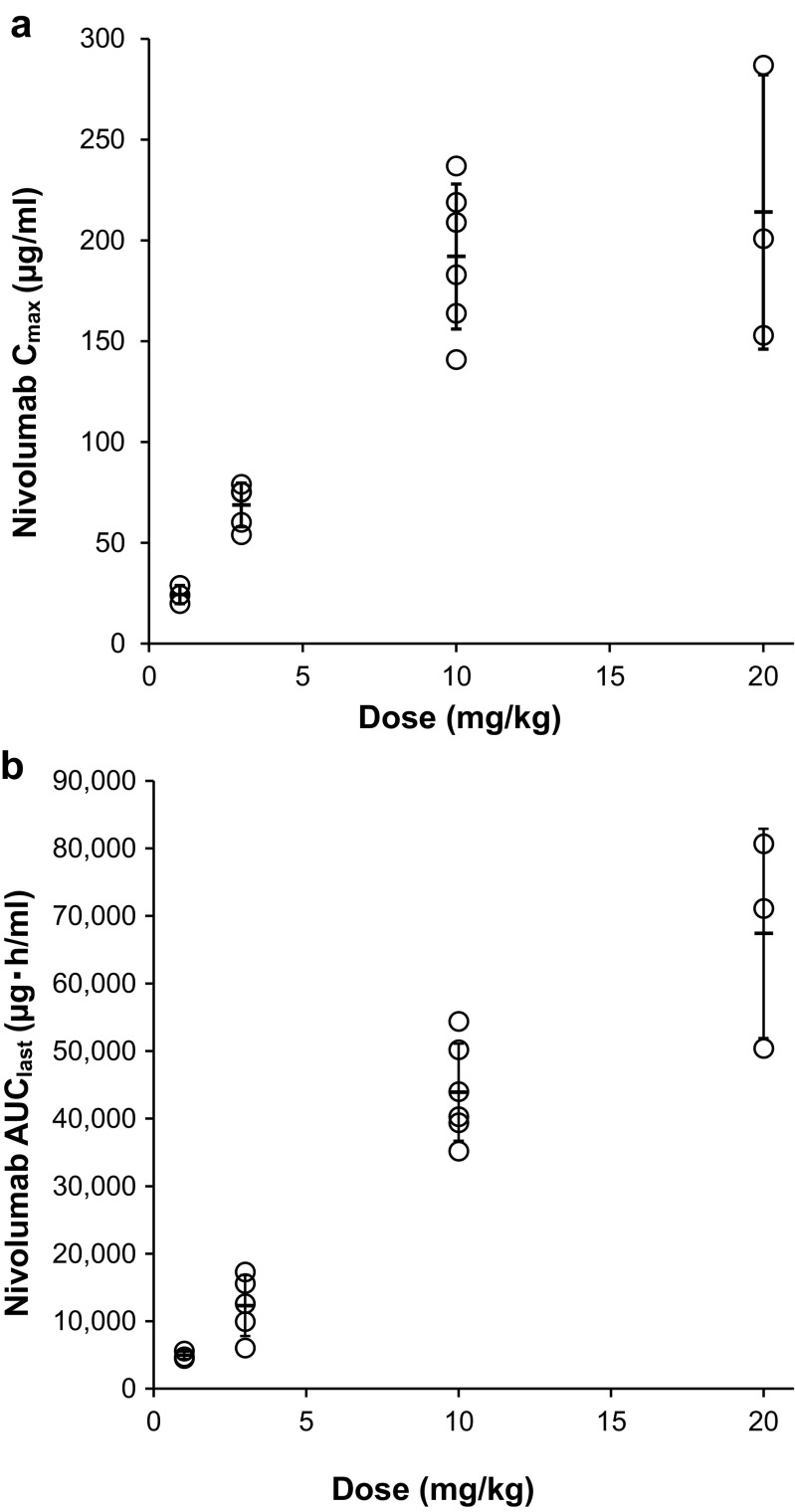
Relationship between doses and C_max_ (**a**) and AUC_last_ (**b**) of nivolumab at single dose of 1 to 20 mg/kg after intravenous continuous administration over 1 h to Japanese malignancy patients

The serum concentration-time profiles of nivolumab after single intravenous infusion at doses of 1–20 mg/kg are shown in Fig. 3. The pharmacokinetic parameters following a single dose of 1–20 mg/kg nivolumab over a period of ≥1 h are presented in Table 3. The mean *t*_1/2_ of serum nivolumab ranged from 13 to 21 days. The AUC_last_ increased in a dose-proportional manner from 1 to 20 mg/kg. While C_max_ increased almost dose-proportionally at nivolumab doses of 1–10 mg/kg, the increase in C_max_ was not dose-proportional at doses of 10–20 mg/kg (Fig. 4).

**Table 3:** Pharmacokinetic parameters of nivolumab after a single intravenous infusion over ≥1 h

	Nivolumab dose			
	1 mg/kg (*n* = 3)	3 mg/kg (*n* = 5)	10 mg/kg (*n* = 6)	20 mg/kg (*n* = 3)
C_max_ (μg/ml)	24.4 ± 4.5	68.8 ± 10.9	192 ± 36	214 ± 68
*t*_max_^a^ (h)	3.0 (1.0–9.0)	1.0 (1.0–3.0)	3.0 (1.0–9.0)	9.0 (3.0–25)
AUC_last_ (μg·h/ml)	4950 ± 580	12,300 ± 4500	43,900 ± 7200	67,400 ± 15,500
AUC_inf_ (μg·h/ml)	8000 ± 1390	20,000 ± 11,300	82,700 ± 18,700	126,000 ± 62,000
*t*_1/2_ (h)	360 ± 10	320 ± 170	520 ± 270	410 ± 230
*t*_1/2_ (day)	15 ± 0	13 ± 7	21 ± 11	17 ± 9
CL (ml/h/kg)	0.127 ± 0.02	0.21 ± 0.152	0.126 ± 0.027	0.206 ± 0.143
V_SS_ (ml/kg)	64.6 ± 6.7	69.7 ± 10.2	83.6 ± 27.4	96.8 ± 12.1

Data are means ± standard deviation

^a^Median (range)

### Efficacy

**Fig. 5:**
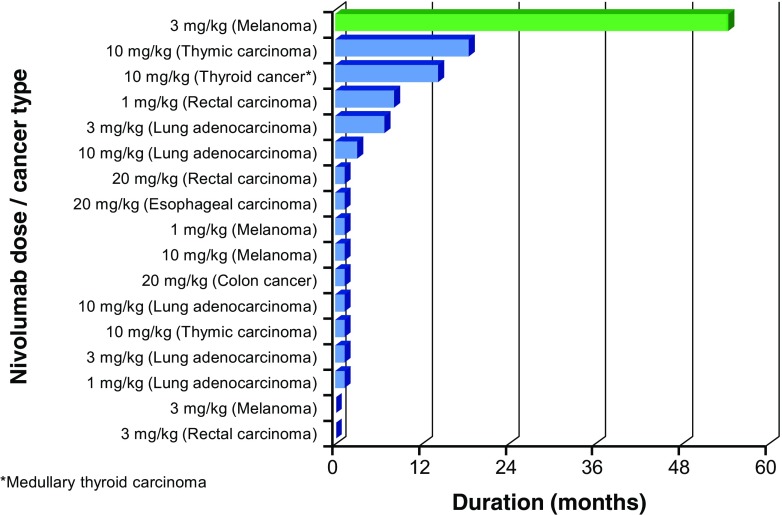
Duration of response in 17 patients in the efficacy population between start and end of the study The melanoma patient (indicated in green) has had a complete response for ≥55 months at time of writing

The best overall responses are shown in Table 4, and the duration of response in each patient is shown in Fig. 5. Based on the RECIST guidelines (v1.0), a CR was observed in one patient (melanoma) in the 3 mg/kg cohort and PR was observed in one patient in each of the 1 mg/kg (colorectal cancer) and 10 mg/kg (medullary thyroid cancer) cohorts. Online Resource Fig. 1 shows the tumor regression in these two patients.

**Table 4:** Best overall response

Nivolumab dose (mg/kg)	Total	CR	PR	SD	PD
1	3	0	1 (RC)	0	2
3	5	1 (Melanoma)	0	1 (NSCLC)	3
10	6	0	1 (Thyroid cancer)	2 (Thymic cancer, NSCLC)	3
20	3	0	0	0	3
Total	17	1	2	3	11

*CR* complete response, *RC* colorectal cancer, *NSCLC* non-small cell lung cancer, *PD* progressive disease, *PR* partial response, *SD* stable disease

#### Correlation of PD-L1 status with clinical response

PD-L1 expression in tumor tissue was evaluated by immunohistochemistry in 11 patients. High PD-L1 expression was defined as a staining intensity relatively equal to that of a positive control (placenta). Low PD-L1 was defined as weaker staining intensity than that of the positive control. A PR was observed in 2/8 patients with high PD-L1 expression. An objective response was not observed in any of the patients with low PD-L1 expression.

### Pharmacodynamics

No remarkable changes were observed in the lymphocyte subset analysis, antigen immune reactivity assays using peripheral blood mononuclear cells, or serum cytokine and immunoglobulin (antibody) levels during the study.

## Discussion

We evaluated the safety, tolerability and pharmacokinetic profiles of single and multiple doses of nivolumab given to 17 Japanese patients with malignant solid tumors in this open-label, dose-escalation study. We also assessed the efficacy and pharmacological activity of nivolumab in these patients.

No DLTs were observed with nivolumab at any dose tested up to 20 mg/kg, and the MTD could not be determined. The latter result is consistent with other studies performed overseas [23–25], although the maximum dose tested was 20 mg/kg in our study compared with 10 mg/kg in the other studies.

ADRs were reported in all patients, but all types of ADRs were previously described in patients treated with nivolumab, and no new safety concerns were raised. Grade ≥3 ADRs (lymphocyte count decreased) were observed in the 3 and 20 mg/kg dose groups in our study (20.0% and 33.3%, respectively). These were transient and the patients recovered without treatment.

Importantly, we found no major differences in the incidences or severities of ADRs among the four dose groups, even after multiple doses of up to 20 mg/kg, thereby confirming that nivolumab is well tolerated at doses of up to 20 mg/kg. Although the small number of patients in the 20 mg/kg cohort means these results should be interpreted with caution, there may be positive implications for outpatient management of ADRs, even at high doses of nivolumab.

The mean *t*_1/2_ of serum nivolumab was 13–21 days after single doses of 1, 3, 10 or 20 mg/kg nivolumab administered over a period of 1 h to Japanese patients with malignant solid tumors. The AUC_last_ of nivolumab increased almost dose-proportionally at doses of 1–20 mg/kg. The C_max_ of nivolumab increased almost dose-proportionally at doses of 1–10 mg/kg, but increased less than dose-proportionally at doses of 10–20 mg/kg. This may have resulted from the wide interindividual variability in C_max_ values in the 20 mg/kg cohort.

The current study included a maximum dose of 20 mg/kg, unlike other published studies of nivolumab, and indeed our own unpublished study, in which we used a maximum dose of 10 mg/kg. It was important to explore the effects of higher doses of nivolumab because the MTD had not been described in prior studies using doses of up to 10 mg/kg, and the possibility of a flat dose was being considered. It was therefore important to confirm the safety of a higher dose of nivolumab, and to determine whether efficacy increased at a higher dose. The latter was not found to be the case, with no responders among the 20 mg/kg cohort, indicating that the efficacy of nivolumab is unlikely to increase with a dose of 20 mg/kg. While our findings are from Japanese patients, who generally have a smaller body mass than Western populations, the pharmacokinetics and safety of nivolumab are apparently unaffected by ethnicity (unpublished data: Nivolumab [Anti-PD-1] BMS-936558 Ethnicity Insensitivity Report; Bristol-Myers Squibb).

Using the RECIST criteria, we observed a CR in one patient (melanoma) in the 3 mg/kg group, and PRs in one patient each in the 1 mg/kg (colorectal cancer) and 10 mg/kg (medullary thyroid carcinoma) groups. The latter result is interesting because, to our knowledge, there are no published data regarding the efficacy of nivolumab in medullary thyroid carcinoma. Further trials of nivolumab in this cancer may therefore be warranted.

As with the other studies mentioned [23–25], we also observed durable responses. In one case, CR was maintained for more than 5 years. Prolonged disease stabilization has also been observed in prior studies of nivolumab [24, 25].

In our study, objective responses were observed in 2/8 (25%) patients (one CR in a patient with melanoma, and a PR in colorectal cancer) with high PD-L1 expression, but CR or PR were not observed in patients with low PD-L1 expression. Similar trials have shown a stronger relationship between response and PD-L1 expression. Topalian et al. observed an objective response (either CR or PR) in 36% of patients with PD-L1–positive tumors and in none with PD-L1–negative tumors [25], while a systematic review of 20 trials in metastatic melanoma, NSCLC, and RCC patients receiving anti–PD-1/PD-L1 antibodies showed a significant decrease (53%) in the risk of mortality in patients with malignant melanoma positive for PD-L1, but no such response relationship was observed for NSCLC or RCC [26]. Therefore, the use of PD-L1 as a possible biomarker clearly requires further investigation.

Anti-nivolumab antibody tests were performed several times, and were positive in two patients in the single-dose phase between study start and study discontinuation, but not during the multiple-dose phase or extended treatment phase. No serious allergic reactions were observed. There were no major differences in AEs between patients positive and negative for anti-nivolumab antibodies (unpublished data: ClinicalTrials.gov [NCT00441337]).

The PDCD1 gene was first discovered by Ishida and colleagues in 1992 [27]. They hypothesized that PD-1 functions as a cell-death inducer, but other possibilities must also be considered. For example, the PD-1 product may be a marker for phagocytosis, or may ‘rescue’ some cells from a dying cell population, or that PD-1 is a byproduct of programmed cell death-induced cells [27]. Blocking PD-1 may help overcome immune resistance to cancer by modulating both innate and adaptive immune responses in tumors.

All patients in this study had advanced refractory solid tumors for which no other therapy had been successful. Tumors develop many resistance mechanisms, including local immune suppression, induction of tolerance, and systemic dysfunction in T-cell signaling [28–31]. Other treatments under investigation for some such tumors include the anti–CTLA-4 antibody ipilimumab, an immune-checkpoint-pathway inhibitor that was effective in advanced melanoma [32–34]. Combinations of these agents may provide greater benefit than each agent alone. Nivolumab combined with ipilimumab showed superior survival benefits in patients with metastatic melanoma compared with ipilimumab alone [35]. Further studies of these new immunotherapeutic agents alone or in combination are vital for the future success of treatment of advanced refractory cancers.

The results of this study should be considered in light of its limitations. In particular, the study enrolled a low number of patients, and variations in clinicopathological features among patients could have influenced the results.

## Conclusions

Nivolumab administered at doses of 1, 3, 10, and 20 mg/kg was associated with good tolerability up to the maximum dose, with no DLTs observed and no MTD defined. In terms of the pharmacokinetics of nivolumab, AUC_last_ was linear at doses of up to 20 mg/kg, and C_max_ was linear at doses of up to 10 mg/kg. Nivolumab achieved CR in one patient and PR in two patients in this Japanese population with advanced refractory malignant solid tumors. Further trials of nivolumab in a larger patient population are warranted. Although we could not determine the MTD, the limited efficacy of nivolumab at 20 mg/kg relative to the lower doses suggests that the maximum dose of nivolumab should be 10 mg/kg in future studies. The dose dependency of the tumor response was not clear. Additional studies might be necessary to establish the recommended therapeutic dose in Japanese patients.

## Electronic supplementary material

ESM 1 (PDF 686 kb)

## References

[1] 2010 Annual summary of monthly vital statistics (round figure), Statistics and Information Department, Ministry of Welfare and Labour. 8–13, 2011, Ministry of Welfare and Labour

[2] Prevalence/mortality/prognosis – 2004 white book for cancer statistics, co-edited by Akira Oshima, Tetsuo Kuroishi, Kazuo Tajima. 201–34, 2004, Shinohara Shuppan Shinsha

[3] Oldham RK (1984). Biologicals and biological response modifiers: fourth modality of cancer treatment. Cancer Treat Rep.

[4] Waldmann TA (2003). Immunotherapy: past, present and future. Nat Med.

[5] Rosenberg SA, Yang JC, Restifo NP (2004). Cancer immunotherapy: moving beyond current vaccines. Nat Med.

[6] Francisco LM, Sage PT, Sharpe AH (2010). The PD-1 pathway in tolerance and autoimmunity. Immunol Rev.

[7] Okazaki T, Honjo T (2006). The PD-1-PD-L pathway in immunological tolerance. Trends Immunol.

[8] Wang J, Okazaki IM, Yoshida T (2010). PD-1 deficiency results in the development of fatal myocarditis in MRL mice. Int Immunol.

[9] Zou W, Chen L (2008). Inhibitory B7-family molecules in the tumour microenvironment. Nat Rev Immunol.

[10] Hamanishi J, Mandai M, Iwasaki M (2007). Programmed cell death 1 ligand 1 and tumor-infiltrating CD8+ T lymphocytes are prognostic factors of human ovarian cancer. Proc Natl Acad Sci U S A.

[11] Hino R, Kabashima K, Kato Y (2010). Tumor cell expression of programmed cell death-1 ligand 1 is a prognostic factor for malignant melanoma. Cancer.

[12] Ohigashi Y, Sho M, Yamada Y (2005). Clinical significance of programmed death-1 ligand-1 and programmed death-1 ligand-2 expression in human esophageal cancer. Clin Cancer Res.

[13] Thompson RH, Kuntz SM, Leibovich BC (2006). Tumor B7-H1 is associated with poor prognosis in renal cell carcinoma patients with long-term follow-up. Cancer Res.

[14] Nomi T, Sho M, Akahori T (2007). Clinical significance and therapeutic potential of the programmed death-1 ligand/programmed death-1 pathway in human pancreatic cancer. Clin Cancer Res.

[15] Nakanishi J, Wada Y, Matsumoto K, Azuma M, Kikuchi K, Ueda S (2007). Overexpression of B7-H1 (PD-L1) significantly associates with tumor grade and postoperative prognosis in human urothelial cancers. Cancer Immunol Immunother.

[16] Iwai Y, Ishida M, Tanaka Y, Okazaki T, Honjo T, Minato N (2002). Involvement of PD-L1 on tumor cells in the escape from host immune system and tumor immunotherapy by PD-L1 blockade. Proc Natl Acad Sci U S A.

[17] Weber JS, D’Angelo SP, Minor D (2015). Nivolumab versus chemotherapy in patients with advanced melanoma who progressed after anti-CTLA-4 treatment (CheckMate 037): a randomised, controlled, open-label, phase 3 trial. Lancet Oncol.

[18] Rizvi NA, Mazières J, Planchard D (2015). Activity and safety of nivolumab, an anti-PD-1 immune checkpoint inhibitor, for patients with advanced, refractory squamous non-small-cell lung cancer (CheckMate 063): a phase 2, single-arm trial. Lancet Oncol.

[19] Motzer RJ, Rini BI, McDermott DF (2015). Nivolumab for metastatic renal cell carcinoma: results of a randomized phase II trial. J Clin Oncol.

[20] Hamanishi J, Mandai M, Ikeda T (2015). Safety and antitumor activity of anti-PD-1 antibody, nivolumab, in patients with platinum-resistant ovarian cancer. J Clin Oncol.

[21] Ansell SM, Lesokhin AM, Borrello I (2015). PD-1 blockade with nivolumab in relapsed or refractory Hodgkin’s lymphoma. N Engl J Med.

[22] Guideline in regard to clinical evaluation for anticancer drugs (PFSB/ELD Notification No. 1101001) (2005), November 1

[23] Brahmer JR, Drake CG, Wollner I (2010). Phase I study of single-agent anti–programmed death-1 (MDX-1106) in refractory solid tumors: safety, clinical activity, pharmacodynamics, and immunologic correlates. J Clin Oncol.

[24] Brahmer JR, Tykodi SS, Chow LQ (2012). Safety and activity of anti-PD-L1 antibody in patients with advanced cancer. N Engl J Med.

[25] Topalian SL, Hodi FS, Brahmer JR (2012). Safety, activity, and immune correlates of anti-PD-1 antibody in cancer. N Engl J Med.

[26] Gandini S, Massi D, Mandalà M (2016). PD-L1 expression in cancer patients receiving anti PD-1/PD-L1 antibodies: a systematic review and meta-analysis. Crit Rev Oncol Hematol.

[27] Ishida Y, Agata Y, Shibahara K, Honjo T (1992). Induced expression of PD-1, a novel member of the immunoglobulin gene superfamily, upon programmed cell death. EMBO J.

[28] Topalian SL, Weiner GJ, Pardoll DM (2011). Cancer immunotherapy comes of age. J Clin Oncol.

[29] Mellman I, Coukos G, Dranoff G (2011). Cancer immunotherapy comes of age. Nature.

[30] Drake CG, Jaffee E, Pardoll DM (2006). Mechanisms of immune evasion by tumors. Adv Immunol.

[31] Mizoguchi H, O’Shea JJ, Longo DL, Loeffler CM, McVicar DW, Ochoa AC (1992). Alterations in signal transduction molecules in T lymphocytes from tumor-bearing mice. Science.

[32] Leach DR, Krummel MF, Allison JP (1996). Enhancement of antitumor immunity by CTLA-4 blockade. Science.

[33] Hodi FS, O’Day SJ, McDermott DF (2010). Improved survival with ipilimumab in patients with metastatic melanoma. N Engl J Med.

[34] Robert C, Thomas L, Bondarenko I (2011). Ipilimumab plus dacarbazine for previously untreated metastatic melanoma. N Engl J Med.

[35] Larkin J, Chiarion-Sileni V, Gonzalez R (2015). Combined nivolumab and ipilimumab or monotherapy in untreated melanoma. N Engl J Med.

