# Supplementary material for: Phase I study of Nivolumab, an anti-PD-1 antibody, in patients with malignant solid tumors
Source: Invest New Drugs. 2016 Dec 8;35(2):207–16. doi: 10.1007/s10637-016-0411-2 (PMC5352798; doi:10.1007/s10637-016-0411-2)
Supplement: Supplementary file 1 — (PDF 686 kb) [file 10637_2016_411_MOESM1_ESM.pdf]

Phase I Study of Nivolumab, an Anti-PD-1 Antibody, in Patients with Malignant Solid Tumors

Noboru Yamamoto[1], Hiroshi Nokihara[1], Yasuhide Yamada[2], Takashi Shibata[1], Yosuke Tamura[1], Yoshitaka Seki[1], Kazunori Honda[1], Yuko Tanabe[1], Hiroshi Wakui[1], Tomohide Tamura[1]

[1]Department of Thoracic Oncology, National Cancer Center Hospital, Tokyo, Japan

[2]Department of Gastrointestinal Oncology, National Cancer Center Hospital, Tokyo, Japan

Corresponding author

Noboru Yamamoto, MD, PhD

Department of Thoracic Oncology, National Cancer Center Hospital

5-1-1 Tsukiji, Chuo-ku, Tokyo 1040045, Japan

E-mail: nbryamam@ncc.go.jp

Online Resource Fig. 1

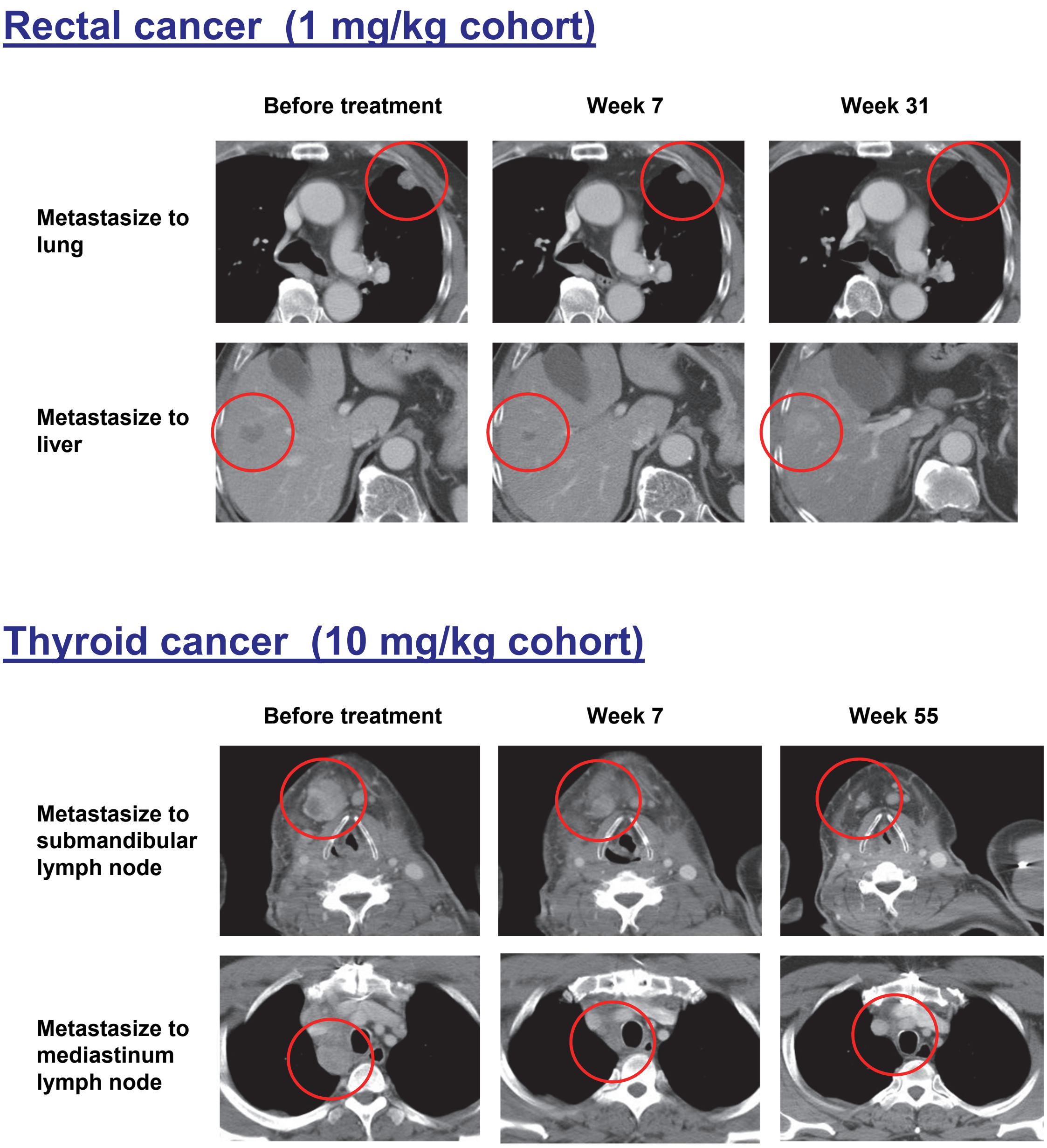

Online Resource Fig. 1 Cancer regression in a patient with rectal cancer treated with 1 mg/kg nivolumab and a patient with thyroid cancer treated with 10 mg/kg nivolumab.
